# Generative Artificial Intelligence Tools: Evaluating Ways to Automate Your SALT (GATEWAYS) Scoring of Alopecia Areata

**DOI:** 10.1111/pde.70195

**Published:** 2026-03-09

**Authors:** Radhika Gupta, Hojjat Salmasian, Michelle Oboite, Sarah Mehlmann, Griffin Stockton Hogrogian, Leslie Ann Castelo‐Soccio, George Cotsarelis, Albert C. Yan

**Affiliations:** ^1^ Perelman School of Medicine University of Pennsylvania Philadelphia Pennsylvania USA; ^2^ Data and Analytics (DnA) Children's Hospital of Philadelphia Philadelphia Pennsylvania USA; ^3^ Dermatology Section Children's Hospital of Philadelphia Philadelphia Pennsylvania USA; ^4^ Division of Dermatology, Children's National Medical Center George Washington University School of Medicine and Health Sciences Washington DC USA

**Keywords:** alopecia, alopecia areata, artificial intelligence, autoimmune disease, clinician reported outcome, Pediatric Dermatology

## Abstract

**Background:**

Alopecia areata (AA) is an autoimmune disease affecting hair follicles that results in nonscarring hair loss. AA impacts 0.1%–0.2% of the United States population, with pediatric patients accounting for 16.0%–27.7% of all cases. The Severity of Alopecia Tool (SALT), a method of quantifying scalp alopecia, helps guide clinical practice and determine response to therapies in clinical trials. Given the emerging role of image‐based assessments of alopecia and growth of multimodal generative artificial intelligence (AI) in dermatology, we aimed to assess the “off‐the‐shelf” ability of a large‐language model, GPT‐4o, to automate the generation of image‐based SALT scores.

**Methods:**

Chart review of patients with AA seen at the Children's Hospital of Philadelphia's Dermatology Clinic was conducted to identify 4‐view images of patients' scalps and provider‐derived SALT scores. One‐hundred‐and‐four 4‐view image sets were de‐identified and provided to GPT‐4o, which was prompted to generate SALT scores. Concordance between GPT‐4o's and providers' scores was determined using intraclass correlation coefficients (ICC) and concordance correlation coefficients (CCC).

**Results:**

ICC and CCC between GPT‐4o and in‐person provider assessments were 0.815 and 0.866. ICC and CCC between GPT‐4o and image‐based provider assessments were 0.833 and 0.817. ICC and CCC between two providers were 0.950 and 0.948. These high levels of concordance were confirmed on Bland–Altman plots.

**Conclusions:**

SALT scoring for AA can be challenging due to provider subjectivity, changing sphericity and growth of patients' scalps, particularly among pediatric patients. Our data show the potential adjunct role that “off‐the‐shelf” generative AI tools may play in SALT scoring without any prior additional explicit training.

## 1 Introduction

Alopecia areata (AA) is an autoimmune disorder affecting hair follicles that results in nonscarring hair loss and has a profound physical and psychological impact on patients [1, 2]. AA affects nearly 0.1%–0.2% of the United States population, with 40% of patients experiencing their first episode of hair loss by age 20 [3, 4]. Treatment options for patients with AA range from intralesional or topical corticosteroids to systemic immunosuppressants, including a recently approved class of medications called Janus kinase inhibitors. The choice of treatment is guided by the progression and severity of AA and commonly defined by the extent of hair loss on the scalp [5]. The Severity of Alopecia Tool (SALT), a clinician‐reported outcome measure, is the primary standardized method of quantifying scalp hair loss in patients with AA.

SALT scores are calculated using the following formula: SALT score = 0.18 × (percentage hair loss on left) + 0.18 × (percentage hair loss on right) + 0.4 × (percentage hair loss on vertex) + 0.24 × (percentage hair loss on occiput), and range in value from 0 (no hair loss) to 100 (total hair loss) [6]. Accuracy in quantifying hair loss via SALT scoring is important for standardization of patient care and for clinical trials; however, physician‐related subjectivity exists, especially in determining the extent of hair loss upon manual inspection, and the additional time needed to quantitatively assess hair loss is a major limitation in clinical practice [7, 8]. A study published by Seol et al. in 2023 found that SALT scores based on retrospective assessment of photos was more clearly correlated with actual surface area than SALT scores based on inspection in the clinic [9]. Additionally, the changing sphericity and growth of patients' scalps among pediatric patients can make SALT scoring challenging for clinicians. To our knowledge, no studies have evaluated the use of generative AI tools to automate or aid in SALT scoring.

In this study, we assess the ability of multimodal generative AI technologies, such as GPT‐4o, to calculate SALT scores of patients' scalps based on de‐identified 4‐view images. Our aim is to determine the concordance between the SALT scores generated by GPT‐4o and those determined by dermatology providers to better understand the role that such tools may play in more accurately assessing hair loss in patients with AA.

## 2 Methods & Materials

### 2.1 Collection of 4‐View Scalp Images

Using a patient list maintained by the principal investigator (AY) of this study, we identified patients with AA who had presented to the Dermatology Clinic at the Children's Hospital of Philadelphia (CHOP). A chart review was conducted to identify those patients with 4‐view (right, left, top/vertex, back/occiput) image sets of their scalps in their medical charts. All image sets (*n* = 104 sets) were redacted and cropped to remove all personal identifiers and associated meta‐data and stored on a secure storage to maintain patient confidentiality. Data masking was conducted to derisk the data, which included replacing medical record numbers with pseudo identifiers, as well as covering the patients' eyes with black boxes in the photographs. Redacted image sets along with the SALT scores determined by their providers at the time of image capture in clinic were recorded (“In‐Person SALT Scores”) (*n* = 79 sets).

### 2.2 Image‐Based Assessment of SALT Scores by Dermatology Providers

Four‐view image sets without existing SALT scores in the chart (*n* = 25 sets) were provided to 2 separate providers in the CHOP Dermatology department via RedCap surveys. Providers were prompted to provide SALT scores for each of the image sets (“Image‐Based SALT Scores”). Of these 25 sets, SALT scores for 2 sets were removed from further analysis due to large discrepancies between the human clinician providers (i.e., 0 versus 100), which were attributed to provider entry error.

### 2.3 Assessment of SALT Scores by GPT‐4o

We used the GPT‐4o model by OpenAI (version 2024‐11–20, accessed via Microsoft Azure) to analyze the redacted photos. This version of GPT‐4o employed by our hospital offers additional features: compliance with data privacy regulations and requirements, robust data encryption and access controls, along with the strong network security features that help ensure user privacy for patient health information (PHI). The data exchanged with the GPT‐4o model in this project was not retained or used for any other purpose, such as for training or validating AI models. For each patient, four photographs (four views: top/vertex, right, left, and back/occiput) were presented, and the AI model was asked through a prompt Figure S1 to determine the view of each image provided (left, right, top, or back) and the percentage of hair loss in each view. The prompt included instructions for the AI to generate the output as a comma‐separated string such as “back,100” or “left,5”. Of the 104 image sets provided, GPT‐4o failed to provide SALT scores for 2 sets of 4‐view images, which were then excluded from further analysis. First, we determined whether GPT‐4o accurately identified the view of each image and calculated the percent correctness for each view. Then, for the remaining 102 image sets, we calculated total SALT scores by using the percentages of hair loss in each view that were determined by GPT‐4o and assigning the correct view to each image.

## 3 Statistical Analysis

To determine the inter‐rater variability between GPT‐4o and providers' SALT scores and between the two providers' SALT scores, concordance correlation coefficients (CCC), intraclass correlation coefficients (ICC (1, 3)), and Pearson correlation coefficients (assuming provider SALT scores are the “gold standard”) were calculated, and Bland–Altman plots were created.

## 4 Results

### 4.1 Assessing GPT‐4o's Ability to Determine the Scalp View and Generate SALT Scores

Overall, GPT‐4o took 1–2 s to process each image provided. Due to the inbuilt content filtering feature of GPT‐4o, the model initially did not return a response for several images. However, once content filtering was turned off, it generated responses for all but two images (2/416) from 2 different 4‐view image sets. Given this, a total of 100 image sets (400 images) were included in our data analysis, after removing the 2 image sets (8 images) with significant discrepancies between provider SALT scores and the 2 image sets (8 images) that GPT‐4o failed to provide SALT scores for. Of the 400 images in total, GPT‐4o correctly identified the scalp view (right, left, top, back) shown in the image 68.8% of the time (275/400). GPT‐4o correctly identified “right” in 68.0% (68/100) of images, “left” in 13.0% (13/100), “top” in 97.0% (97/100), and “back” in 97.0% (97/100). We used the estimated hair loss percentage from GPT‐4o to calculate the SALT scores, regardless of whether GPT‐4o was able to identify the scalp view correctly.

### 4.2 Inter‐Rater Variability in SALT Scores

**FIGURE 1:**
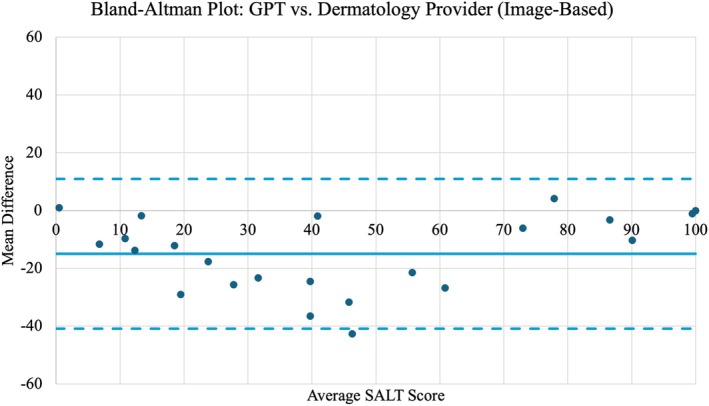
Bland–Altman Plot Comparing GPT and Image‐Based Human Assessments of SALT Scores.

**FIGURE 2:**
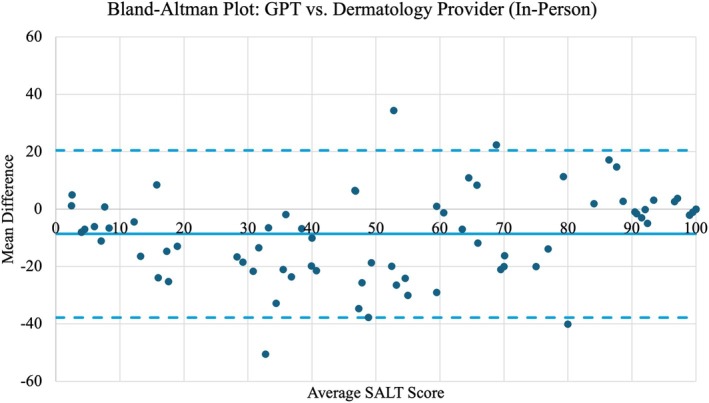
Bland–Altman Plot Comparing GPT and In‐Person Human Assessments of SALT Scores.

**FIGURE 3:**
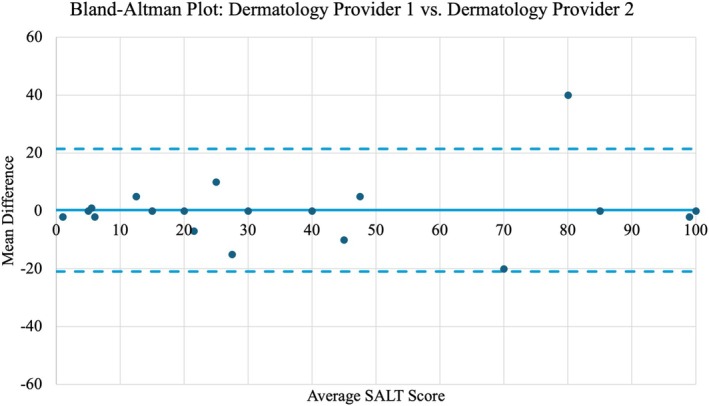
Bland–Altman Plot Comparing Two Independent Human Assessments of SALT Scores.

We compared the SALT scores generated by GPT‐4o and in‐person provider assessments (*n* = 77 sets) and found the CCC, ICC, and Pearson correlation coefficient to be 0.866, 0.815, and 0.903, respectively. The corresponding Bland–Altman plot (mean difference of −9) is shown in Figure 1. We then compared the SALT scores generated by GPT‐4o and image‐based provider assessments (*n* = 23 sets) and found the CCC, ICC, and Pearson correlation coefficient to be 0.817, 0.833, and 0.917, respectively. The corresponding Bland–Altman plot (mean difference of −15) is shown in Figure 2. Lastly, we compared the SALT scores between two independent image‐based provider assessments (*n* = 23 sets) and found the CCC and ICC to be 0.948 and 0.950, respectively. The corresponding Bland–Altman plot is shown in Figure 3.

## 5 Discussion

In this study, we assessed the ability of GPT‐4o to calculate SALT scores based on de‐identified 4‐view images of patients' scalps. We found that GPT‐4o identified the view incorrectly in 32% of the cases (e.g., for an image labeled as “left,” the model may have identified it as the “right” view). The model correctly labeled “left” views in only 13% of the cases, making more left/right errors than other errors. This could suggest that GPT‐4o misinterpreted the orientation of the patients' scalp in the images, given that a prior study demonstrated inconsistencies in GPT's ability to assess anatomical regions in radiological images. Going forward, it may be helpful to provide GPT‐4o with additional detail in the initial prompt on how the image was obtained (e.g., “a dermatology provider took 4 separate images of a patient's scalp (top/vertex, back/occiput, left, right) while facing the patient during an in‐person clinic visit”) or to directly provide the view of the scalp in the image. As secure, privacy‐compliant generative AI platforms become more widely available, they will be able to assess identifiable 4‐view images, which could help improve AI's ability to determine the view and overcome the content filtering issue that we faced. Furthermore, future studies should explore how image characteristics (e.g., resolution, orientation) and patient‐specific characteristics (e.g., Fitzpatrick skin type, hair type) affect GPT's ability to reliably calculate SALT scores.

Pairwise comparisons of SALT scores produced by providers (in‐person and image‐based) and GPT‐4o showed high concordance suggesting that GPT‐4o produced scores that agreed closely with the “standard” rater (an experienced human clinician provider). Based on CCC values, SALT scores produced by GPT‐4o agreed more closely with in‐person provider scores; however, based on ICC values, GPT‐4o scores agreed more closely with image‐based provider scores. This was further supported by the Bland–Altman plots (Figures 1 and 2) with mean difference lines close to 0, suggesting no significant systematic bias between GPT‐4o and providers. Though, the negative values of the mean difference lines indicate that GPT‐4o tends to calculate a higher percentage of hair loss compared to providers.

Comparison of SALT scores produced by dermatology providers based on images alone showed high concordance. This was further supported by the Bland–Altman plot (Figure 3) with a mean difference line of 0, suggesting no significant systematic bias between the raters. There were also few to no points outside the limits of agreement, suggesting that the raters agreed well on most measurements of SALT scores. While prior studies have raised concern about provider variability in SALT scores, the concordance between the two providers in our study could be a function of working closely within the same Pediatric Dermatology hair clinic. This further underscores the need for standardization in SALT scoring across dermatology and primary care providers who may be taking care of pediatric patients with alopecia areata.

Our approach utilized an off‐the‐shelf generative AI that can automate the SALT scoring process without requiring prior training. The system performed well and demonstrated significant concordance with human expert clinicians who specialize in pediatric hair disorders. The performance of the off‐the‐shelf GPT‐4o compares favorably to a proprietary AI system built on a Unet with EfficientNetB4 base model reported by Nguyen et al. Their study demonstrated ICC scores between 0.95 and 0.97, indicating excellent correlation. We suspect that future updates to existing off‐the‐shelf models will undoubtedly improve their performance further [8].

While our study is limited by single center data collection and data from only one generative AI platform, the high concordance between GPT‐4o and experienced Pediatric Dermatology hair clinic providers highlights the potential adjunct role that off‐the‐shelf generative AI tools like GPT‐4o may play in helping providers, patients with alopecia areata, and patients' families track hair loss over time, even in the absence of consistent access to dermatologic care. Furthermore, AI‐based tracking of alopecia over time may serve as a source of motivation for patients to adhere to therapies—Chan et al. reported on a patient who decided to continue with treatment after noticing that the SALT scores generated by AI demonstrated clear progress [9]. These tools may also aid dermatologists in more accurately determining patient eligibility for therapies that require certain SALT score thresholds to be met for approval by insurance companies.

## Funding

Salary support for the first author of the study (R.G.) was provided by a Pediatric Dermatology Research Alliance Emerging Investigator Grant.

## Ethics Statement

The Institutional Review Board of the Children's Hospital of Philadelphia provided an IRB exemption for this study (Protocol #24–033713). Informed consent was not obtained given that data masking was performed.

## Conflicts of Interest

R.G., consults for Cabaletta Bio. G.C., consults for Lilly. A.Y., consults for Johnson & Johnson, Pierre Fabre, Regeneron‐Sanofi, Verrica, and is an investigator for Arcutis, Aucta, Boehringer Ingelheim, Timber (Leo). S.M., G.H., H.S., L.C., and M.O., have no disclosures.

## Supporting information


**Data S1:** pde70195‐sup‐0001‐Supinfo.docx.

## Data Availability

The data that support the findings of this study are available from the corresponding author upon reasonable request.
