# Supplementary material for: Generative Artificial Intelligence Tools: Evaluating Ways to Automate Your SALT (GATEWAYS) Scoring of Alopecia Areata
Source: Pediatr Dermatol. 2026 Mar 9;43(4):824–8. doi: 10.1111/pde.70195 (PMC13422994; doi:10.1111/pde.70195)
Supplement: Supplementary file 1 — Data S1: pde70195‐sup‐0001‐Supinfo.docx. [file PDE-43-824-s001.docx]

**Supplementary Figures**

**Supplementary Figure 1**: Prompt Provided to GPT-4o to Generate SALT Scores

“The Severity of Alopecia Tool (SALT) score for alopecia areata is calculated by evaluating four views of the scalp (top/vertex, right, left, and back/occiput) and determining the percentage of hair loss in each area.

These percentages are then combined using a weighted formula to calculate the overall SALT score, which ranges from 0 (no hair loss) to 100 (complete scalp hair loss).

The SALT score is calculated using a formula that takes four variables, each representing the percentage of hair loss on one view of the scalp (left, right, top or back).

Your job is to analyze a scalp photo I have provided and determine the response to each of the following questions.

1. Which of the 4 scalp views (left, right, top or back) is shown in the photo? The response should be only a single word, such as "left" or "back".

2. What percentage of hair loss appears to have occurred in this view? The response should be only a single number between 0 and 100 percent, without the percentage sign.

You should return those responses in a comma-separate format, such as back,100 or left,5.”
